# Current Ratio and Stability Issues of Electronically Enhanced Current Transformer Stimulated by Stray Inter-Winding Capacitance and Secondary-Side Disturbance Voltage

**DOI:** 10.3390/s22197565

**Published:** 2022-10-06

**Authors:** Peter Zajec

**Affiliations:** Faculty of Electrical Engineering, University of Ljubljana, Tržaška 25, SI-1000 Ljubljana, Slovenia; peter.zajec@fe.uni-lj.si; Tel.: +386-14-76-8479

**Keywords:** current transformer, current ratio, inter-winding capacitance, trans-conductance amplifier, disturbance rejection

## Abstract

Electronically enhanced current transformers (EECT) have gained much interest in power quality assessment. Their magnitude and phase angle error, which mainly relates to the properties of the ferromagnetic materials used, the impedance of the secondary load, and the inter-turns capacitance, are thoroughly analyzed. In contrast, the capacitance between the windings, i.e., inter-winding capacitances and their limiting effects on EECT operation, are rarely analyzed in detail—in particular, no details on the control design of the assisting electronic unit, its tuning recommendations, or both are provided. In this paper, the capacitive coupling between indication and compensating winding of EECT with simplified feedthrough construction is analyzed thoroughly in terms of current ratio error and stability of the implemented configuration of the trans-conductance amplifier. The preliminary assumption about the adverse effect of the inter-winding capacitance shunting both ends of the original amplifier, composed of two series-connected inverting amplifier stages, was confirmed and resolved within a modified amplifier with the help of a simplified simulation model and was experimentally proven with measurements on a custom-built EECT prototype. Furthermore, the analyzed phenomena were linked to trans-conductance amplifier parameters, explicitly with its compensating networks, and summarized in their design guidelines. Throughout the paper, the EECT features obtained with original and modified amplifier designs are compared with the plain composite current transformer to demonstrate the benefits of the modified amplifier, especially its robustness against inter-winding capacitance variations.

## 1. Introduction

In recent years, the usage of precise instrument current transformers (CT) [1] is no longer confined merely to the top laboratory and calibration facilities. Instead, they are gaining momentum due to the widespread application of grid-tied converters, where precise metering of power, energy and power quality (PQ) assessment is essential [2]. To comply with accompanying standards, e.g., IEC 60044-1 and IEEE C57.13, the representative CT features, such as the nominal accuracy and frequency bandwidth, must be accomplished across a wide measuring range, frequently ranging between 0.1% and 120% of the nominal current.

The bandwidth in PQ assessment is usually limited to power grid frequency up to its 20th harmonic, although it can extend up to 9 kHz [2,3]. In general, the CT accuracy can be increased either by ferromagnetic core quality improvements or active techniques that modify the magnetic quantities in a compensating manner (denoted as electronically enhanced CT (EECT) or electronically assisted CT). An example of the first approach is use of composite ferromagnetic cores. Combining two cores with different magnetic properties is beneficial compared to homogenous cores as the high permeability value of the first core guarantees high accuracy at low measuring currents. In contrast, the higher flux density saturation of the second core assures the operation at the upper end of the current range [4]. Even though the composite CTs can achieve a remarkable accuracy improvement, their weight and dimensions become a significant disadvantage if higher accuracy, higher throughput power, or both are required. In this regard, and at the expense of a more sophisticated design and higher cost, the EECT efficiently outperforms the composite CT.

As reported in [5], the EECT’s ratio errors of less than 10 × 10^−6^ in both magnitude and phase can be achieved at power frequencies. In either case, such errors can be attained only with sandwich-like core assembly, which significantly adds to the manufacturing costs [6,7]. Another cost driver is a multi-stage structure suggested in [5,8,9]. In either case, the generalized EECT requires at least two cores. Regardless of the control concept, the assisting electronics must generate and feed a compensating current to nullify the current difference between the primary and the secondary current. Thus, the core magnetization should be correspondingly adapted—either by feeding the compensating current directly through the secondary load [10] or indirectly through the additional compensating winding [11,12].

The construction-related current errors associated with ferromagnetic parameters, leakage inductance, secondary-side impedance requirements, and stray capacitance between turns are widely known and analyzed in detail [11,13,14]. On the other hand, the stray inter-winding capacitances and their limiting effects on CT’s operation are rarely analyzed in detail [15,16,17,18,19,20]. In particular, no details on the control design of the assisting electronic unit, its tuning recommendations or both are provided. Furthermore, no assessment of the dynamical control performance is carried out.

This paper discusses the abovementioned issues regarding the calibration requirements for direct connected poly-phase energy meters with current and voltage circuits permanently closed in each phase, i.e., with no means to open the link between voltage and current circuits. The specific application requires that each kWh meter’s current branch be isolated from a common calibration source. In order to reduce human-related errors, the CT’s transformation should be done with the fixed turn ratio (1:1) over the entire measuring range (Table 1).

In general, the demanded accuracy surpasses the capacity of traditional and composite current transformers. Thus, the usage of electronically enhanced current transformers (EECT) is mandatory. However, except for a small nominal frequency range (45 Hz up to 65 Hz), all other requirements surpass those of PQ. In particular, the 60 VA nominal power is, by a rule, up to two decades higher than the power rating of EECTs used in precise metering equipment ranging below 5 VA. Nevertheless, the most challenging constraint is that even under 150 mA the specified error must be below the minimum limit of 0.05% to be commercially competitive.

This paper aims to provide an in-depth analysis of the EECT sensitivity to the stray inter-winding capacitances and to evaluate their impacts on current ratio error and control instability. In particular, the original amplifier consisting of two inverting amplifier stages is found to be much more prone to instabilities caused by unintentional positive feedback formed by the parasitic inter-winding capacitance. As a result, the overall frequency response of the amplifier could become underdamped, generating high-frequency oscillations superimposed to the nominal frequency component (45 Hz up to 65 Hz) on the secondary side of the EECT. In order to minimize the impact of parasitic capacitance, this paper proposes a modified amplifier, with the first stage being changed to a non-inverting one. Apart from swapping the ends of the compensating winding, no further change is required. The effects of these modifications and robustness against inter-winding capacitance variations are thoroughly compared for both variants of the EECT and for demonstration purposes also with a composite current transformer to highlight the advantages of the amplifiers and identify their potential weaknesses.

## 2. Construction and Modelling of the EECT

The magnetizing current (*I_0_*) of the transformer causes a difference between the primary (*I_p_*) and the secondary current (*I_s_*). They differ in magnitude and phase, both being frequency dependent. Considering a 1:1 turn ratio, the same applies to the current ratio defined as

(1) $${\underline{H}}_{I}=\frac{{\underline{I}}_{p}}{{\underline{I}}_{s}}=\left|{\underline{H}}_{I}(f)\right|\cdot e^{j\delta_{I}(f)}.$$

**Figure 1:**
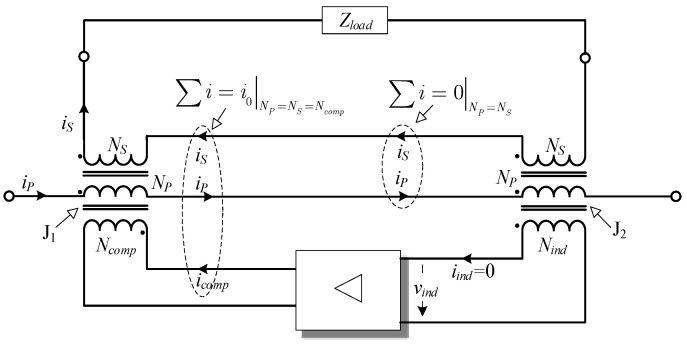
The EECT’s conceptual scheme.

A design with inherent isolation between the assisting electronic and the primary and secondary winding enables a so-called feedthrough transformer construction. Regardless of the control concept, the assisting electronics should generate and feed a compensating current (*i_comp_*) to nullify the current difference. At least two cores are needed, as is evident from the generalized concept of EECT shown in Figure 1.

**Figure 2:**
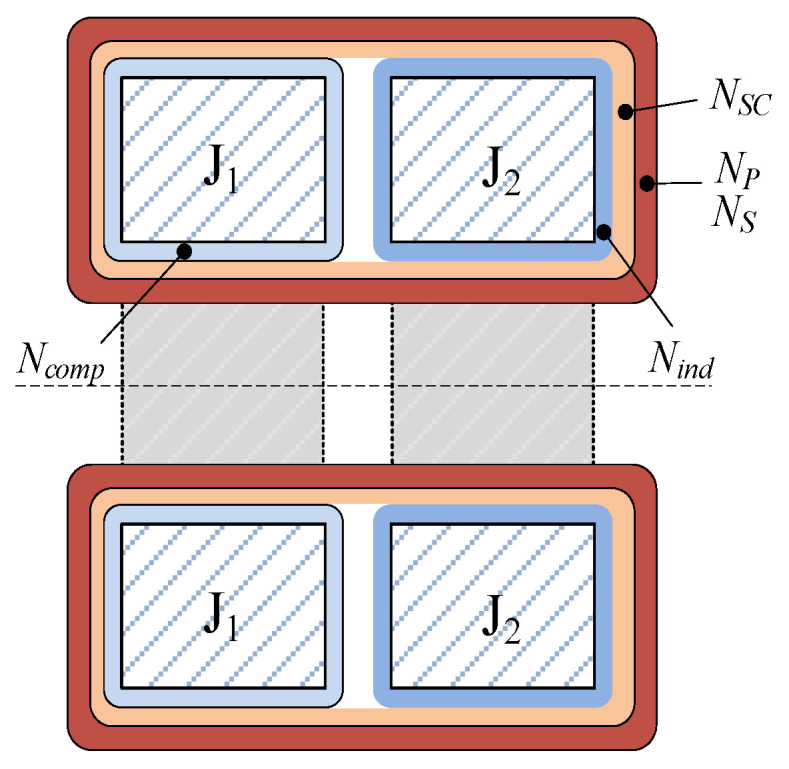
Cross-section view of cores with indicated windings.

In the custom-built prototype, both cores of toroidal shape have equal inner and outer diameters (60 mm × 100 mm) and height (35 mm). The cross-section view in Figure 2 shows that the cores are positioned side by side to achieve optimal feedthrough construction. The compensation core (J_1_) is made of nanocrystal material with *N_comp_* = 50, while the indication core (J_2_) is made of Ni-Fe alloy and is wound with *N_ind_* = 400. Both cores are manufactured by OMEM S.p.A, Italy. Before placing the primary and secondary winding (*N_p_* = *N_s_* = 6), cores are stacked and are uniformly wound with an additional “short-circuit” winding (*N_SC_* = 500). Its purpose is to protect the employed amplifiers against overvoltage if the secondary load impedance is above the nominal or when the secondary circuit becomes open-circuited during the operation. Subsequently, the primary and secondary windings are bifilarly and uniformly wound around the entire diameter, assuring a magnetic coupling between J_1_ and J_2_.

In addition to magnetic coupling provided by primary and secondary winding, the cores are coupled with the electronic unit through windings *N_ind_* and *N_comp_*. Nevertheless, since the amplifier (Figure 1) has infinite input impedance, the J_2_ magnetization is assumed to depend exclusively on primary and secondary currents. In order to make them equal, the electronic unit needs to magnetize the main core (J_1_) with *I_comp_* = *I_0_* (assuming *N_comp_* = *N_p_* = *N_s_*).

The studied EECT core and winding structures are reasonably simplified compared to known sandwich core structures [6,7] due to priority cost optimization, as one EECT per phase must be provided in the calibration plant for each kWh meter being tested, and the count of which may extend up to 40. Consequently, any shielding derived from the sandwich core structure or additional added layer is omitted from the design. Nevertheless, from the control perspective, the prototype is generally equivalent to the solutions reported in [10].

### EECT Current Error Derivation

**Figure 3:**
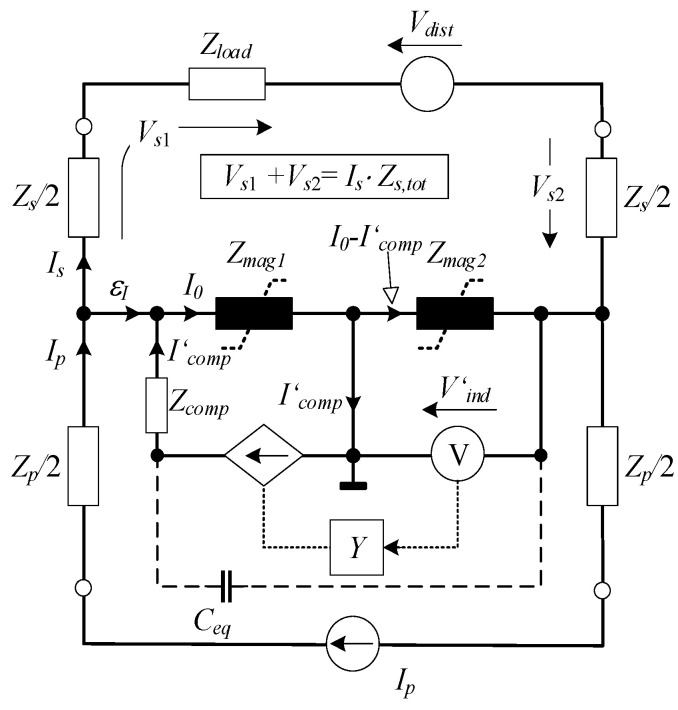
EECT’s equivalent circuit.

Assisting electronics can be analyzed more thoroughly by applying the standard transformer equivalent circuit (Figure 3). The non-linear magnetizing impedances of cores are denoted as *Z_mag_*_1_ and *Z_mag_*_2_.

For simplicity, the leakage impedances of primary and secondary winding split among both cores are assumed to be equal to *Z_p_* and *Z_s_*, respectively. A total impedance of the secondary side (*Z_s,tot_*) corresponds to the sum of a nominal load impedance (*Z_load_*) and the value of *Z_s_*. Parameters and quantities of compensation and indication circuits are transposed to the primary side and annotated with (‘). The assisting electronics behave as a voltage-dependent current source, i.e., trans-conductance (TC) amplifier with a frequency response *Y*. A disturbance voltage source (*V_dist_*) is included in the secondary circuit to derive a generalized analysis. The *C_eq_*, equivalent capacitance, is discussed in detail in the subsequent chapters.

Referring to EECT’s equivalent circuit, the current error

(2) $${\underline{I}}_{p}-{\underline{I}}_{s}={\underline{\varepsilon }}_{I}={\underline{I}}_{0}-{\underline{I}}_{comp}^{\prime }$$

Can only be nullified when *I*’*_comp_* perfectly matches the magnetizing current as already stated. In order to support the secondary current, the sum of induced voltages in both cores should be in equilibrium with the total voltage drop in the secondary circuit

(3) $${\underline{I}}_{0}({\underline{Z}}_{mag1}+{\underline{Z}}_{mag2})-{\underline{I}}_{comp}^{\prime }{\underline{Z}}_{mag2}={\underline{I}}_{s}{\underline{Z}}_{s,tot}^{}-{\underline{V}}_{dist}.$$

The compensation current (*I*’*_comp_*) fed by the TC amplifier is proportional to its frequency response and to the voltage detected across the indication winding

(4) $${\underline{I}}_{comp}^{\prime }=\underline{Y}({\underline{I}}_{0}-{\underline{I}}_{comp}^{\prime }){\underline{Z}}_{mag2}.$$

Applying (3) and (4) to (2), the current error can be derived

(5) $${\underline{\varepsilon }}_{I,EECT}=\frac{{\underline{I}}_{s}{\underline{Z}}_{s,tot}^{}-{\underline{V}}_{dist}}{{\underline{Z}}_{mag1}+{\underline{Z}}_{mag2}+{\underline{Z}}_{s,tot}^{}+\underline{Y}\cdot {\underline{Z}}_{mag1}{\underline{Z}}_{mag2}}.$$

Assuming *V_dist_* = 0 V and *Y* = 0, i.e., an omitted TC amplifier, the (5) describes the error of a composite current transformer, stating that the error can only be small when the total secondary impedance is negligible. In contrast, the magnetizing impedances should be as high as possible. Due to the presence of the secondary load with its nominal non-zero value, the selection of high-permeability magnetic materials has, in this circumstance, a substantial impact on the attained current error of composite CT.

The benefit of the TC amplifier is evidently revealed by comparing the error of the composite CT with (5) considering *Y* ≠ 0.

(6) $${\frac{{\underline{\varepsilon }}_{I,EECT}}{{\underline{\varepsilon }}_{I,composite}}|}_{{\underline{V}}_{dist}=0}=\frac{{\underline{Z}}_{mag1}+{\underline{Z}}_{mag2}+{\underline{Z}}_{s,tot}^{}}{{\underline{Z}}_{mag1}+{\underline{Z}}_{mag2}+{\underline{Z}}_{s,tot}^{}+\underline{Y}\cdot {\underline{Z}}_{mag1}{\underline{Z}}_{mag2}}=\frac{1}{1+\underline{Y}\cdot \frac{{\underline{Z}}_{mag1}{\underline{Z}}_{mag2}}{{\underline{Z}}_{mag1}+{\underline{Z}}_{mag2}+{\underline{Z}}_{s,tot}^{}}}.$$

The (6) reveals that the EECT’s error can be substantially decreased if condition *Y* · *Z_mag_*_1_ · *Z_mag_*_2_ >> *Z_mag_*_1_+ *Z_mag_*_2_ + *Z_s,tot_* is fulfilled. In order to comply with the condition, both magnetic impedances must possess high impedance, i.e., high permeability. However, as the decrease of *Z_mag,x_* can be compensated for by increasing the gain (*Y*), i.e., the number of suitable magnetic materials expands. Nevertheless, a clear distinction between both cores exists. Namely, when the condition *I’_comp_* = *I_0_* is satisfied, the power to the secondary side is transmitted ideally only through the main (J_1_) core since the induced voltages across auxiliary core windings tend to be infinitely small. That could lead to the false conclusion that J_2_ quality is irrelevant to achieving EECT’s target accuracy. In contrast, by rearranging (4)

(7) $${\underline{I}}_{comp}^{\prime }={\underline{I}}_{0}\frac{\underline{Y}\cdot {\underline{Z}}_{mag2}}{1+\underline{Y}\cdot {\underline{Z}}_{mag2}},$$

It becomes evident (7) that for a given *Y*, the error can be cancelled out (*I’_comp_* = *I_0_*) only if |*Z_mag_*_2_| approaches infinity. As a result, the magnetic properties and dimensions of EECT’s cores, e.g., height, usually differ as |*Z_mag_*_2_| > |*Z_mag_*_1_| is preferred.

**Figure 4:**
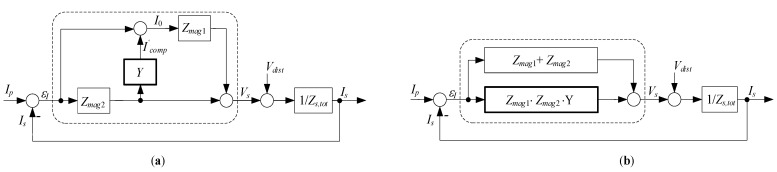
EECT’s functional diagram: (**a**) drawn in accordance with (2), (3) and (4); (**b**) rearranged.

The control aspects of the EECT can be better understood by examining its functional diagram (Figure 4a) drawn in accordance with (2), (3), and (4). The *V_s_* denotes the total voltage induced to sustain voltage drops in the secondary circuit. An even more obvious distinction between composite CT and EECT functions is demonstrated in the rearranged diagram of Figure 4b.

## 3. Description of the Electronic Unit

The electronic unit, behaving as a voltage-dependent current source, helps to minimize the order of the system as the *Z*’*_comp_* can be neglected, i.e., considered as part of the high-impedance current source. Since a dedicated current source feeds the primary current, the *Z_p_* can also be neglected.

**Figure 5:**
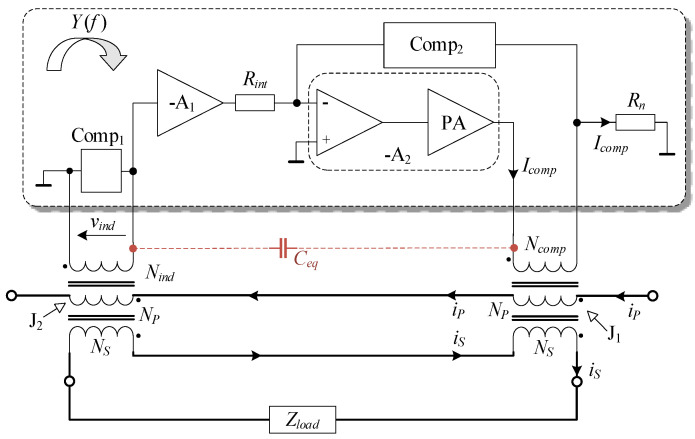
The conceptual scheme for assisting electronics.

Referring to (4), the *Y* is of complex quantity with high magnitude, i.e., gain. As a result, a two-stage amplifier is proposed, allowing more freedom to customize its compensation networks. Both stages are developed as inverting topologies utilizing generic operational amplifiers (Figure 5). However, the preferred high gain is dominantly attained by the second stage—designed as a composite amplifier with inherent current feedback, provided by compensation network Comp_2_. In the output section of the composite amplifier, a subordinate power amplifier (PA) with higher current capability is implemented to provide compensation current up to 200 mA.

In a steady-state operation, the DC component of compensation current should be maintained at a negligibly small value to prevent core saturation. Thus, small offset voltages in the amplifying chain should only experience a small DC gain provided by compensation network Comp_2_. Conversely, the same network must provide a high gain in the frequency range from 45 Hz to 65 Hz—keeping in mind to prevent setting the roll-off frequency of the *Y* frequency response too close to 45 Hz, as this may cause the phase angle to become frequency dependent. As a compromise, the Comp_2_ should provide a large magnitude (gain) and preferably a flat frequency response (*Y*) starting from DC up.

Due to the non-linear behavior of PA load, consisting of compensating winding inductance and the *R_n_*, as a rule, higher harmonics exist in compensating current, which requires the bandwidth of the amplifier chain to exceed the nominal frequency range of 45 Hz to 65 Hz. Subsequently, a larger PA output voltage is preferred—and thus, a higher gain is needed in the closed-loop chain to feed the current’s higher harmonics through a predominantly inductive impedance. In order to avoid PA’s saturation, stability issues, and other compromises linked to high gain, another compensating network (Comp_1_) is placed in parallel with the indication winding and the input of the first stage amplifier—thus virtually shunting the *Z_mag_*_2_. The choice of *Z_mag_*_2_ placement can be better understood by referring to (7). Namely, the zero phase error between *I_p_* and *I_s_*, i.e., *I’_comp_* = *I_0_*, can be merely obtained if the phase shift introduced by the *Y* and the inductive character of *Z_mag_*_2_ in *Im*{*Y* · *Z_mag_*_2_} is compensated to obtain

(8) $$∡\left(\varphi_{Y}+\phi^{*}{}_{mag2}\right)=0.$$

where ϕ^*^_mag__2_ stands for compensated phase shift.

### 3.1. Stray Inter-Winding Capacitances

**Figure 6:**
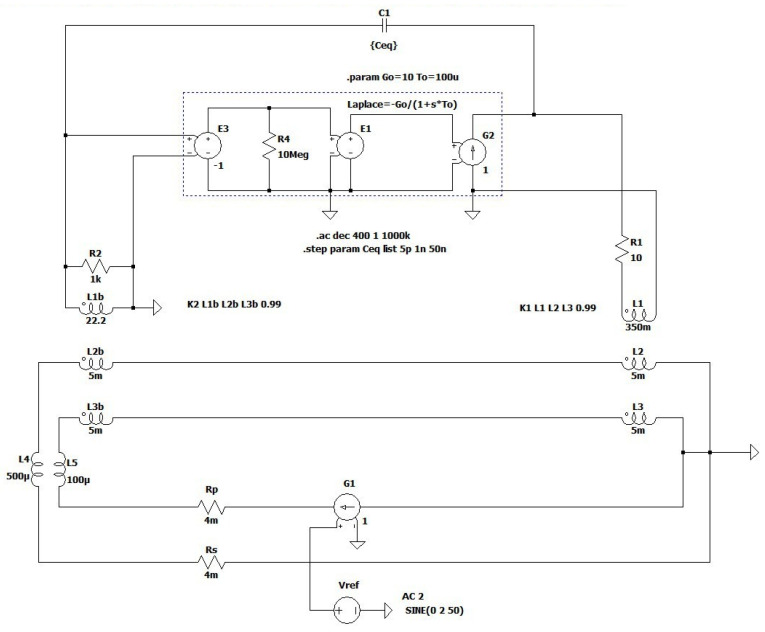
Equivalent EECT scheme with simplified control and compensating networks of the original TC amplifier.

As emphasized in the introduction, the importance of stray inter-winding capacitances is regularly addressed superficially, providing no details on the controller design. The capacitance *C_ps_* between primary and secondary winding is commonly assumed to be uncritical. In contrast, the stray capacitances (*C_pi_*, *C_si_*, *C_ci_*) of primary, secondary, and compensating winding, coupled with the indication winding, cannot be neglected from the analysis despite their small values (some tens pF). In this paper, their effects are combined and represented with an equivalent *C_eq_*. This simplification is valid since voltage drops on leakage impedances are negligible. Namely, in contrast to the *C_ps_*, the *C_eq_* is coupled to the node of high impedance, i.e., the A_1_ input. In terms of control theory, the *C_eq_* virtually shunts the input and output of the TC amplifier, thus introducing an additional feedback path. This can change its feedback response (*Y*), consequently causing its instability due to a decreased magnitude and phase safety margin [22]. The EECT’s stability is generally defined by the product of all subunits transfer functions that compose a control loop (Figure 6), i.e., by open-loop gain.

In the following text, a simplified LTspice model (Figure 6) of the EECT with the original TC is implemented to intuitively study the adverse effects of the parasitic capacitance *C_eq_*, which is design-based and not part of the dedicated feedback loop of the second stage of the TC amplifier. The A_1_ amplifier is modelled as a voltage-dependent voltage source, whereas the PA’s output stage is approximated with a dependent current source. Their responses are simplified with a first-order transfer function. In practice, the Comp_2_ feedback structure comprises a series resistor-capacitor circuit connected in parallel with another resistor. Contrary to TC’s first-order simplification, the applied circuit above 100 kHz introduces an extra zero in the TC transfer function to compensate for phase lags attributed to other unexpected parasitic effects within the PCB design and imperfect shielding. In addition, the compensation network Comp_1_ is purely resistive. Extra impedances inserted in the primary and secondary circuits represent inductances and resistances caused by the cabling harness.

With *C_eq_* neglected, the Nyquist stability criterion is fulfilled by tuning the feedback network Comp_2_, which dominantly defines the PA’s transfer function—and consequently the EECT’s response (*H_I_*, *H_dist_*) in frequency and time domain. However, since the capacitance *C_eq_* bypasses both inverting amplifier stages, it forms an extra, unintentional feedback loop. Through it, any voltage change at the output of the second stage reflects at its inverting input with the opposite sign, forming positive feedback. Because its operation is reversed to second-stage negative feedback, the magnitude and phase limits are reduced, resulting in an under-damped response due to increased open-loop gain of the TC amplifier in a narrow frequency range (specified in literature as bump or response peaking). Its intensity and frequency range change with both complex feedbacks. In addition to negative feedback, the intensity of the positive is defined by a frequency depended on relationship quantified by the impedance *C_eq_* and resistance (mainly defined by compensating network Comp_1_) seen at the input of the first stage of the amplifier, where they form a capacitive-resistive voltage divider.

**Figure 7:**
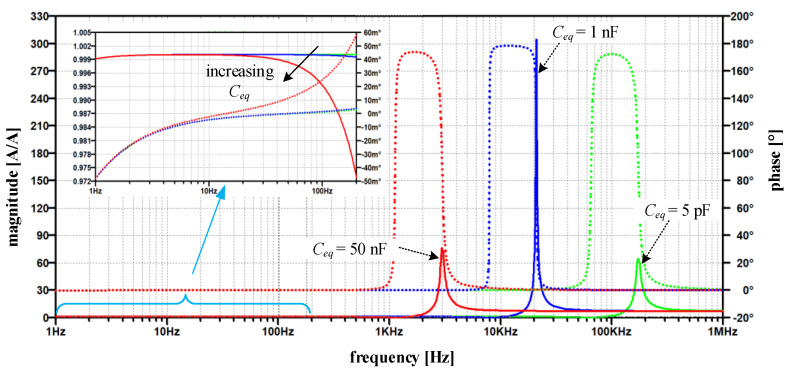
Magnitude and phase of the current ratio *H_I_*—original circuit (magnitude-solid line, phase—dotted line); for varying *C_eq_* = {5 pF, 1 nF, 50 nF} with detailed *H_I_* between 1 Hz and 200 Hz.

Their result is seen in Figure 7, which highlights the change in the current ratio’s magnitude (solid line) and phase (dotted line) at different simulated values of *C_eq_*.

Its smallest value (5 pF) is attributed to the estimated parasitic capacitance between connecting wires of both windings. The 1 nF is a more realistically estimated capacitance and relates both to the printed circuit design of the TC amplifier and, above all, to the relative position of both respective windings. In practice, this capacitance depends on the distance between the cores and their height-to-width ratio. In order to assess the *C_eq_* effects on EECT performance and to test robustness against parameter variations, 50 nF was chosen as the upper value. Although the maximum peaking in Figure 7 occurs at *C_eq_* = 1 nF it should be noted that this does not necessarily represent the global peak.

**Figure 8:**
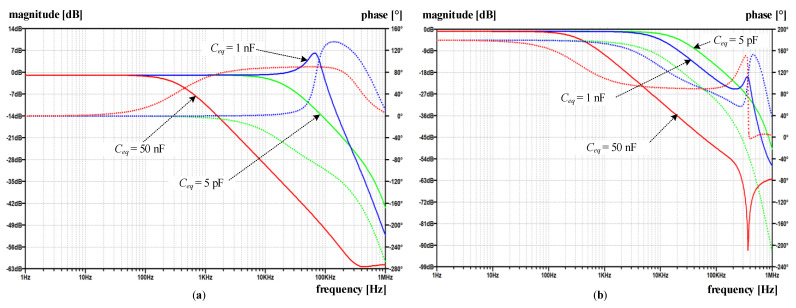
Simulated frequency response *Y* (magnitude-solid line, phase—dotted line) with original and modified amplifier for varying *C_eq_*: (**a**) original amplifier; (**b**) modified amplifier.

Practically up to 1 kHz the magnitude ratio |*H_I_*| is close to 1 and phase error in 10^−3^ range. (Figure 7). As the capacitance increases, the magnitude ratio modifies moderately in the range where the fundamental component of compensating current is expected (detail in Figure 7). On the contrary, at higher frequencies, we observe a distinct frequency band with a significant increase in the magnitude ratio, which passes to lower frequencies as the capacitance increases. Notably, the maximum peak does not occur at the maximum or the minimum capacitance, but rather at its in-between value. This behavior is inherent to positive feedback intensity, i.e., its complex gain explained above relative to the cut-off frequency of the TC amplifier (1/*T*_0_ in Figure 6)—which is mainly defined by compensating network Comp_2_. The *Y* frequency response shows the same behavior, where its peaking occurs at *C_eq_* = 1 nF rather than at 5 pF or 50 nF (Figure 8a).

**Figure 9:**
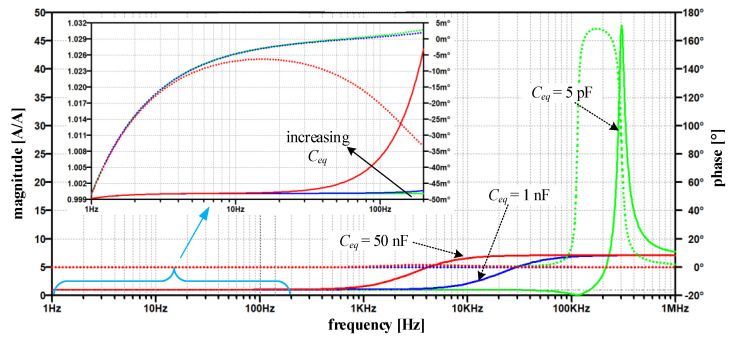
Magnitude (solid line) and phase (dotted line) of the current ratio *H_I_*—modified circuit for varying *C_eq_* = {5 pF, 1 nF, 50 nF } with detailed *H_I_* between 1 Hz and 200 Hz.

The above-discussed positive feedback origin can be straightforwardly verified by modifying the input stage (Figure 6) to a non-inverting one. Nevertheless, the closed-loop phase relationship must be preserved; that is why the direction of compensating current needs to be altered, e.g., by swapping the ends of the compensating winding. The obtained results in Figure 9 show that the modified topology predominantly changes the high-frequency behavior of the current ratio. In particular, compared to the original amplifier topology (Figure 7), the high-frequency peak is reduced by more than 42 times for the *C_eq_* = 1 nF, except in the case of the smallest value of *C_eq_*, where the reduction is less than 30%. In contrast to the original circuit, the most significant observation is that current ratio peaks are limited as soon the *C_eq_* is high enough.

Nevertheless, no significant improvement nor deterioration is gained in the low-frequency range, where |*H_I_*| moves away from 1 in both cases as *C_eq_* increases. At this point, it is worth noting that the reported *H_I_* summarizes the small-signal AC responses implemented on a linearized and simplified circuit. Thus, the demonstrated behavior is expected to deviate in practical circumstances due to the non-linearity and saturation introduced by PA and the *B-H* magnetizing curve. Regardless of these simplifications, and without further analysis, the significantly smaller current ratio gain (Figure 9) obtained at higher *C_eq_* reduces the presence of high frequency and high-amplitude secondary currents (compared to *C_eq_* = 5 pF and particularly to the original amplifier in Figure 7). Thus, in the modified amplifier, *C_eq_* forms an additional negative feedback loop, which in turn does not cause the subsequent instability of the TC amplifier, but even helps in its frequency compensation, which is typical for multipath (negative) feedback loops [23,24]. As a result, no additional TC amplifier damping via any of its compensating networks is required.

### 3.2. Disturbance Rejection Capability

Whenever the length of the secondary side harness connecting the EECT with the load is oversized, the intermittence of the nearby circuits can disrupt the current relationship in the EECT. Therefore, any unintended voltage or current source must be considered to warrant the desired accuracy. In this paper, the secondary side disturbance source *V_dist_* emulates the impact of induced voltage caused by the alternating magnetic fields that couple to a possibly oversized secondary harness. Its impact can be assessed in the form of a rejection ratio derived from (5)

(9) $$\left|{\underline{H}}_{dist}\right|=\left|\frac{{\underline{I}}_{s}}{{\underline{V}}_{dist}}\right|=\left|\frac{1}{{\underline{Z}}_{mag1}+{\underline{Z}}_{mag2}+{\underline{Z}}_{s,tot}^{}+\underline{Y}\cdot {\underline{Z}}_{mag1}{\underline{Z}}_{mag2}}\right|.$$

Note that the primary current is independent of secondary side disturbance since an ideal current source is placed in the primary winding

**Figure 10:**
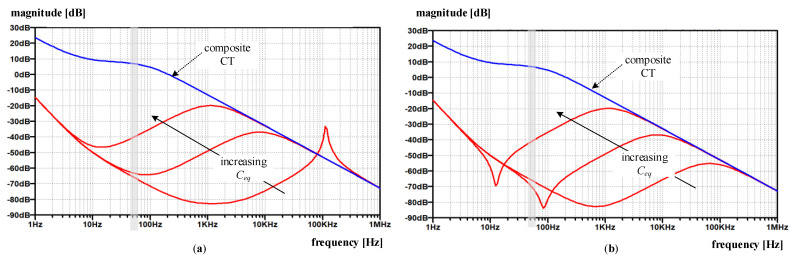
Disturbance rejection capability |*H_dist_*|*_composite CT_* (blue line) vs |*H_dist_*|*_EECT_* (red lines) for varying *C_eq_* = {5 pF, 1 nF, 50 nF}: (**a**) original amplifier; (**b**) modified amplifier.

Compared to the composite CT (Figure 10), the electronic unit improves the rejection ratio almost across the whole frequency range, as can be expected (9). Especially for the original circuit (Figure 10a), one can observe similarities with |*H_I_*| frequency response in respect of more or less narrower frequency bands where the rejection becomes insufficient, or even smaller than what is achieved with a composite CT. Nevertheless, the rejection within the nominal frequency range is sufficiently large and generally decreases with increasing frequency as long as *C_eq_* is not too large. This behavior again underlines the importance of *C_eq_* during the design process-even when an electronic unit with a modified configuration is used (Figure 10b).

### 3.3. Compensating Networks Guidelines

As can be seen from the qualitative analysis of the EECT, the influence of the *C_eq_* regarding the deviation of the current ratio and the attenuation capability *V_dist_* is noticeably favorable in the modified configuration of the two-stage amplifier circuit. In addition, both compensation circuits have been identified as crucial components that significantly affect both frequency responses and must respectively meet the following qualitative conditions:

- In frequency bandwidth extending up to the 20th harmonic of the maximum value of nominal frequency (65 Hz), the TC amplifier gain (*Y*) must be considerably large with a negligible phase shift—both quantities are primarily subjected to Comp_2_;
- To substantially decrease the EECT’s magnitude error (6), the following *Y* · *Z_mag_*_1_ · *Z_mag_*_2_ >> *Z_mag_*_1_+ *Z_mag_*_2_ + *Z_s,tot_* must be fulfilled;
- To attain negligible phase error, the imaginary part of *Y*· *Z_mag_*_2_ must be zero. As *Z_mag_*_2_ is inductive, the Comp_1_ is mandatory to compensate for its phase shift;
- Due to differences in throughput power, the *Z_mag_*_2_ > *Z_mag_*_1_ is preferred.

## 4. Experimental Results

**Figure 11:**
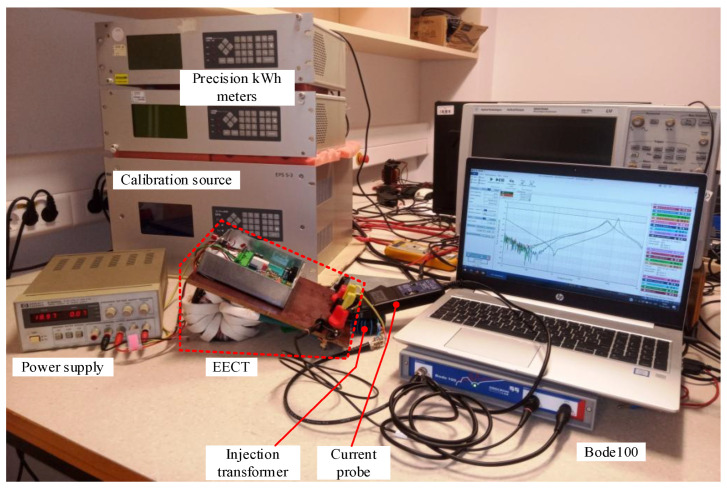
EECT prototype during *H_dist_* measurement. Measuring set-up for |ε_I_| (behind EECT).

The EECT prototype seen in Figure 11 was used to verify the findings. Note that if not explicitly emphasized, all measurements in the following are taken on optimized compensation networks guaranteeing that the current error is within specification limits. The measurements of a custom-built EECT used for testing direct connected poly-phase meters were performed with dedicated energy-based measuring equipment to verify the findings. In particular, two precise electronic kWh-meters employing the time-division multiplier (TDM) approach with nominal 0.01% accuracy were used. Their voltage channels were connected to the same voltage generator.

In contrast, the current channel of the first kWh-meter was connected to the primary circuit in series with the current generator. The second one was installed in the secondary circuit of the tested EECT. Both generators are part of a calibration power supply that can independently adjust their magnitude and phase displacement. As the applied concept of the kWh-meters cannot distinguish between magnitude and phase error, the latter was qualitatively checked by performing measurements at power factor (*PF*) equal to 1 and at ±0.5, i.e., at ±60 degrees displacement between voltage and current generator.

**Figure 12:**
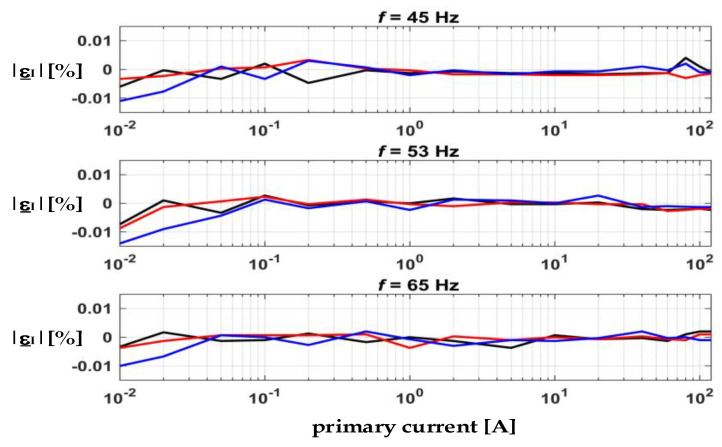
Current error |ε_I_| in % at different frequencies and *PF*: 0.5 capacitive (blue), 1 (red), 0.5 inductive (black).

Before each measurement, both current channels were exposed to identical conditions to obtain the calibration table in the entire current range and at different power factors. That was necessary to take into account the differences inherent to kWh-meters. Subsequently, the measurement, with current channels attached as mentioned above, was repeated at the same measuring points. The results obtained by considering the calibration data are shown in Figure 12.

### 4.1. The Y Frequency Response Measurement

The frequency response *Y* is generally measured by the voltage injection method without opening the control loop [25]. However, its results are valid as long as the impedance Middlebrook’s criteria is obeyed—stating that the impedance seen by the injection generator needs to be zero at one and infinite at the other connecting point. At first glance, the point between the output of A_1_ and the coupling resistor *R_int_* seems the most suitable if only the output impedance of A_1_ was not comparable in magnitude to *R_int_* and dependent on frequency. Another reason for discarding this type of measurement is that during the frequency sweep, the non-linearity involved in the closed loop affects the measurements as the amplitude of the input signal is not under control.

Instead, the frequency response was measured in an open loop configuration with compensating winding detached from the TC amplifier. Its load, i.e., secondary side impedance, was substituted with its transposed value, i.e., with a 277 mΩ resistor. To preserve the magnetic properties in J_2_ similar to operating ones, the magnitude of the injected signal, attached to the A_1_ input, was kept reasonably small. The measurement was performed by the Vector Network Analyzer—Bode100 [26]. In addition to *V_ind_*, the *I_comp_* was captured as a voltage drop across *R_n_* and consequently applied to input channels of the Bode100.

**Figure 13:**
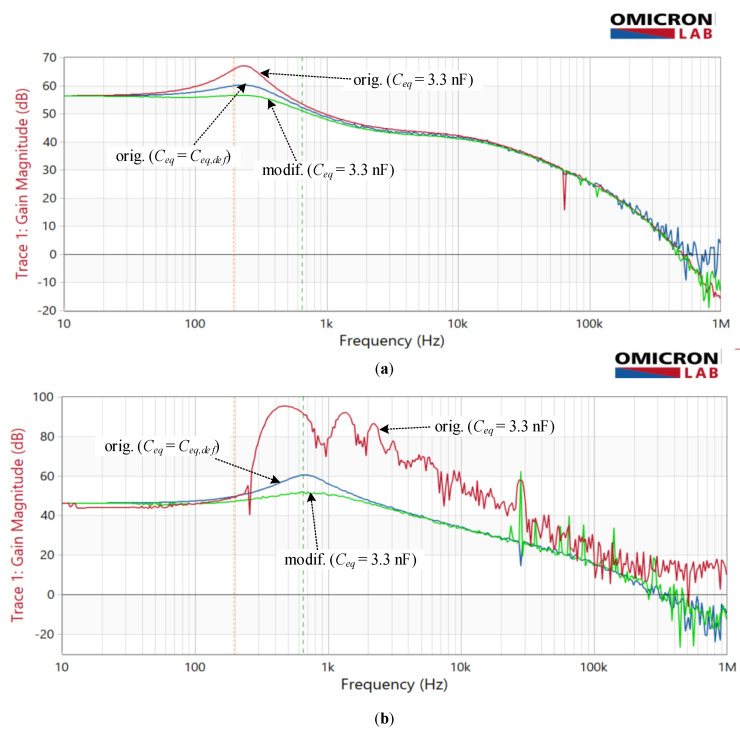
Measured frequency response *Y* with original and modified amplifier in regard to *C_eq_*: (**a**) optimized Comp_2_; (**b**) non-optimized Comp_2_.

The frequency characteristics measured on both amplifier configurations with optimized compensation networks can be seen in Figure 13a. The responses in Figure 13a demonstrate the impact of purposely increased capacitance *C_eq_* on both amplifier configurations. The modified amplifier’s resonant peak is reduced compared to the measurement taken at the default *C_eq_* value. In contrast, it is exceeded in the original amplifier configuration. The default value *C_eq,def_* = 125 pF, defined by design, was assessed with RLC meter HP4274A by measuring the capacitance between indicating and compensation winding with their ends short-circuited.

A similar comparison was then made with the adapted compensation circuit Comp_2_ so that the amplitude and phase margin of the TC was reduced. Consequently, the original amplifier’s response (blue line in Figure 13b) demonstrates a relatively higher peak, as the low-frequency gain is smaller than in Figure 13a. With this change, the increased capacitance *C_eq_* has an entirely different impact on both amplifier configurations. The operation of the modified amplifier remains stable, whereas the original one becomes severely unstable at higher frequencies. It shows a significant gain increase (Figure 13b) around and above the resonant peak. This measurement should nevertheless be taken only as informative since, as during the frequency sweep, the periodic oscillations emerged at the output of the amplifier circuit and consequently overloaded the Bode100 input channels. This, however, did not occur in the case of the modified circuit.

The overloading issue with the measuring equipment is also why measurements were not performed with *C_eq_* = 1 nF, which among a set of simulated values, shows the highest response peaking, but instead with 3.3 nF resulting in less severe overloading.

### 4.2. Disturbance Rejection Measurement

The rejection capability was also measured using the Bode100. In order to emulate a secondary voltage disturbance, its generator was attached via an additional coupling transformer in the secondary circuit. The secondary voltage and current were then captured by a voltage and current probe (TA189 PICO) and fed into the Bode100 input channels. During the measurement, the primary winding was opened to provide a constant primary current, thus emulating the primary current source and assuring the resemblances with the theoretical backgrounds in Section 3.2.

**Figure 14:**
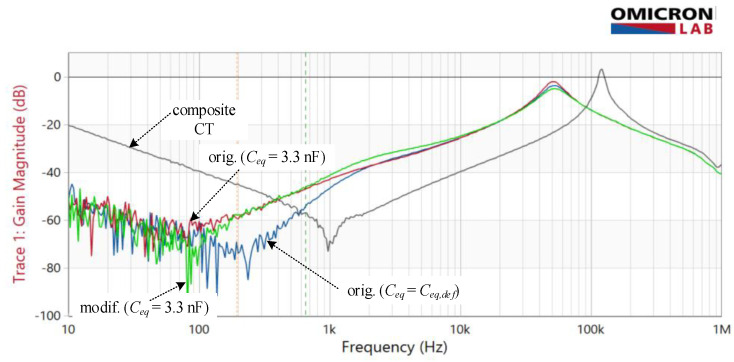
Measured disturbance rejection—*H_dist_* of composite CT (grey) vs EECT with original amplifier with default *C_eq_* (blue), original amplifier with increased *C_eq_* (red) and modified amplifier with increased *C_eq_* (green).

The composite transformer’s attenuation (Figure 14) was measured as a starting point to distinguish the impacts caused by core construction from those resulting from the electronic unit. A continuous increase in attenuation is noticeable for both amplifier configurations up to 1 kHz when it starts to decrease again. The obtained attenuation improvement is approximately 40 dB over the nominal frequency range regardless of whether the default value of *C_eq_* is increased or not. Above 100 Hz, the effect of increasing *C_eq_* is more evident in the form of a reduction in the attenuation of both amplifier configurations compared to *C_eq_* = *C_eq,def_*.

### 4.3. The Experimental Summary

Experimental results (Figure 13 and Figure 14) show a high degree of similarity with the simulation results, despite being obtained on a simplified model of the TC amplifier and the electrical and magnetic properties of the two magnetic components (J_1_ and J_2_). Due to the lack of exact parameters and primarily due to the neglected non-linearity of the magnetic circuit, the results do not match perfectly. In addition, the applied calibration source does not represent an ideal primary current source, as assumed in analysis, and its parameters are neither frequency independent. As a result, the most extensive disagreement is evident at high frequencies of *H_dist_* where the response of amplifiers significantly deviates from the composite amplifier, unlike in the simulations, where their attenuations match.

In addition, the non-linearities, as mentioned above, increase the inaccuracy of the simulation prediction and the complexity of the optimization process beyond the default small-signal analysis. That is why the control loop has been, as a result, optimized solely based on experimental knowledge. A potential upgrade of the simulation model would improve the agreement of the results, but it would be beyond the scope and aim of this paper. Nevertheless, we estimate that the accuracy of the simplified simulation is satisfactory, which is also confirmed by the experimental results.

## 5. Discussion

In this paper, the capacitive coupling between indication and compensating winding of electronically enhanced current transformer with simplified feedthrough construction is analyzed thoroughly in terms of current ratio error and stability of the implemented TC amplifier. In this regard and compared to other publications, the paper highlights the importance of the implemented amplifier configuration. The preliminary assumption about the adverse effect of the inter-winding capacitance shunting both ends of the amplifier composed of two series-connected inverting amplifier stages was confirmed with the help of a simplified simulation model and was experimentally proven by measurements on a custom-built EECT prototype. Furthermore, the analyzed phenomena were linked to TC amplifier parameters, explicitly with its compensating networks, and summarized in their design guidelines.

Despite the greatly simplified simulation model of the EECT, the simulation predictions agree with practical measurements to a large extent. Noticeable deviations, especially those above the nominal frequency range, are mainly attributed to ferromagnetic core modelling that neglects their non-linearity and the first-order approximation of A_1_ and PA frequency responses. The results reveal that modified amplifier configuration with the non-inverting input stage is far more superior regarding *C_eq_* variation. This is evident from *Y* response measurement (Figure 13), demonstrating how much more the original configuration is prone to instability in particular when non-optimized compensating network Comp_2_, which gives rise to the roll-off frequency of the *Y*, is implemented. Note that the amplifier reconfiguration does not require any other costly modifications except straightforward swapping of either indication or the compensation winding ends.

In contrast to *Y*, which has a proven impact on the stability of EECT, the significance of the *H_dist_* measurement at frequencies that are a decade or two higher than the nominal frequency requires additional comment. In both cases, the broad measuring range is far from being irrelevant. Namely, in a specific application, a dedicated switch-type calibration source imposes current through the primary winding, composed of the fundamental, e.g., 50 Hz and high-frequency components, usually in the kilohertz region. Despite their small level, they should be sufficiently attenuated on the secondary side. The secondary side of the EECT likewise is subjected to the presence of high-frequency currents. In this case, their generation can be associated with likely unstable operation of EECTs being part of the implemented precise electronic kWh-meters attached in the primary and secondary circuits. Thus, the disturbance rejection capability is vital to prevent instability from spreading from one system to another.

Although we avoided using dedicated shielding layers to protect the indicating and compensating windings against the dV/dt effects in the primary and secondary circuit, the shielding effect was secured by a tightly wound short-circuit winding grounded with one end to the reference potential of the electronic unit. Of course, the shield’s capability is not perfect due to the non-negligible impedance at higher frequencies, but it’s better than nothing—especially since no particular winding machine or even manual work is required.

## Figures and Tables

**Table 1:** Technical requirements, adapted according to [21].

Technical Parameters	Requirements		
Current range	10 mA up to 120 A		
Nominal frequency	45 Hz up to 65 Hz		
Ratio	1:1		
Power rating	Max. 60 VA at 120 A, max. load voltage 0.5 V over whole current range		
	**Current range**	**Ratio error (%)**	**Phase angle error (min)**
Accuracy	1 mA ≤ *I* ≤ 10 mA	1	50
	10 mA ≤ *I* ≤ 25 mA	0.5	20
	25 mA ≤ *I* ≤ 150 mA	0.2	10
	150 mA ≤ *I* ≤ 120 A	0.05	3
